# Spontaneous Emergence of Legibility in Writing Systems: The Case of Orientation Anisotropy

**DOI:** 10.1111/cogs.12550

**Published:** 2017-10-10

**Authors:** Olivier Morin

**Affiliations:** ^1^ Minds and Tradition Research Group Max Planck Institute for the Science of Human History

**Keywords:** Orientation anisotropy, Oblique effect, Symmetry, Cultural evolution, Neural recycling

## Abstract

Cultural forms are constrained by cognitive biases, and writing is thought to have evolved to fit basic visual preferences, but little is known about the history and mechanisms of that evolution. Cognitive constraints have been documented for the topology of script features, but not for their orientation. Orientation anisotropy in human vision, as revealed by the oblique effect, suggests that cardinal (vertical and horizontal) orientations, being easier to process, should be overrepresented in letters. As this study of 116 scripts shows, the orientation of strokes inside written characters massively favors cardinal directions, and it is organized in such a way as to make letter recognition easier: Cardinal and oblique strokes tend not to mix, and mirror symmetry is anisotropic, favoring vertical over horizontal symmetry. Phylogenetic analyses and recently invented scripts show that cultural evolution over the last three millennia cannot be the sole cause of these effects.

## 1 Introduction

Written signs are created by human brains to be read by human brains. Their shape can instruct us about two issues central to the field of cultural evolution: the influence of cognitive constraints on cultural forms (Sperber & Hirschfeld, 2004) and the origins of functional complexity in culture (Tennie, Call, & Tomasello, 2009). The visual appearance of most scripts fits basic constraints of the human visual system (Changizi & Shimojo, 2005; Changizi, Zhang, Ye, & Shimojo, 2006; Dehaene, 2010). The topology of writing reproduces the patterns of occlusion seen in nature (Changizi et al., 2006). It is widely assumed that such adaptations have been produced by a process of “cultural selection” (Changizi et al., 2006; Dehaene, 2010; Dehaene & Cohen, 2007; Pelli, Burns, Farell, & Moore‐Page, 2006) for two reasons. First is the view that functional complexity in cultural forms is likely to arise from cumulative cultural evolution, not from universal cognitive biases (Boyd, Richerson, & Henrich, 2013). Second, it has been argued that cultural universals are advantageously explained by weak biases amplified by iterated learning (Thompson, Kirby, & Smith, 2016). This paper investigates the fit between visual constraints and cultural forms, and the role of cultural evolution in creating legible scripts. While previous cross‐cultural studies have focused on their topology, this study considers the orientation of strokes in letters.

Several lines of evidence suggest that the two cardinal orientations should dominate in a legible script. Horizontal and vertical lines are easier to recognize, discriminate, and memorize: There is an “oblique effect” in perception in a great variety of species (Appelle, 1972; Mach, 1886). This bias has innate roots in humans (Leehey, Cook, Brill, & Held, 1975). It constitutes an adaptation to the conformation of both natural and human‐made environments: Horizontals and verticals are overrepresented in both (Girshick, Landy, & Simoncelli, 2011). Studies in neuro‐aesthetics suggest that cardinal lines are not only merely easier to process but also aesthetically valued (Latto, Brain, & Kelly, 2000) and extremely prevalent in art and heraldry (Latto & Russel‐Duff, 2002).

Individual letters arguably gain in legibility when they do not combine cardinal with oblique strokes. This minimizes the chances of finding acute angles in letters. Studies in visual search show that recognition of a line is improved when the angular distance between target and distractors is maximized (Foster & Westland, 1995; Wolfe, Friedman‐Hill, Stewart, & O'Connell, 1992). Cardinal lines, in addition to being more accurately perceived than oblique ones, are better detected when masked by cardinal lines of the opposite orientation (Li, Thier, & Wehrhahn, 2000; Sekuler, 1965). In addition to these perceptual advantages, right angles also tend to be copied more faithfully than acute ones, at least by children (Bremner & Taylor, 1982; Davis, Bruyn, & Boyles, 2005). As most of the scripts studied here were hand‐copied by several generations of child learners, a bias favoring right angles would have had a multiplied impact on the shape of letters. It was predicted that cardinal strokes should tend to be found in letters containing other cardinal strokes (and vice versa): a “separation effect.” The Latin script, for instance, has more purely cardinal letters (E) or purely oblique ones (W), but fewer mixed letters (K) than chance would predict.

Written characters should be anisotropic in one last respect: symmetry. Our brains are attuned to vertical (as opposed to horizontal) symmetry (Mach, 1886; van der Helm, 2015; see Brekle, 1994 on the expected effects on writing). Vertically symmetric objects (like faces or standing vertebrate bodies) are a fixture of most humans’ visual world. It is, thus, unsurprising that vertical‐symmetrical shapes are easier to detect and memorize than their horizontal counterpart (Rossi‐Arnaud, Pieroni, Spataro, & Baddeley, 2012; Wenderoth, 1994). Infants as early as 12 months old exhibit an attentional preference for them (Bornstein, Ferdinandsen, & Gross, 1981). The dominance of vertical symmetry is evident in the Latin script: letters like A and T are twice as frequent as E‐ or B‐like letters.

The contribution of cultural evolution to these three forms of legibility (cardinality, separation, vertical symmetry) was measured in two ways. One consists in asking whether extinct scripts are less legible than the ones currently in use (“cultural selection”). A script's legibility and its survival may share similar causal underpinnings. Demography is a plausible candidate. Widely spoken languages have been claimed to be easier to learn than less spoken ones (Lupyan & Dale, 2010). If widely diffused scripts were under increased pressure to be more legible, then the more legible scripts would be less likely to go extinct. My second measure of cultural change reflects change inside a given cultural lineage: whether the scripts that branched out from other scripts, as independent offshoots of a continuing script or as continuations of an extinct one, became more legible than their ancestor (“cultural transformation”).

## 2 Methods

These predictions were tested by analyzing 116 scripts, all taken from the ISO 15924 inventory of scripts (ISO 2013), and standardized by the Unicode consortium (Unicode 1994) (see online supplementary materials, henceforth SM, SM 1, SM 2.1, SM 2.13). Twenty of the 116 scripts were also analyzed on manuscript documents to make sure that my results were not due to a bias brought in by the various designers of Unicode scripts. For each script, three measures were taken (when possible).

### 2.1 Cardinality

The scripts were coded by reporting, for each letter, the number of horizontal (distant from the 180° axis by less than 10°), vertical (less than 10° away from 90°), and oblique lines (all the rest), and averaging this value over all letters in the script (SM 2.2). Cardinal orientations as defined here thus cover 22% of the compass (80/360°). For each script, the average number of cardinal lines per letter (“cardinality”) was computed by two raters, who agreed on their measurement (ICC = 0.970, *n* = 116 scripts). There was also high agreement between cardinality ratings produced for Unicode versions of a script and for manuscript versions (ICC = 0.935, *n* = 20). (All ICCs reported in this paper are two‐way mixed intra‐class correlations assessing absolute agreement on the average value of a measurement taken at script level, computed using the icc function of the irr package in R.)

### 2.2 Separation of obliques and cardinals

**Figure 1:**
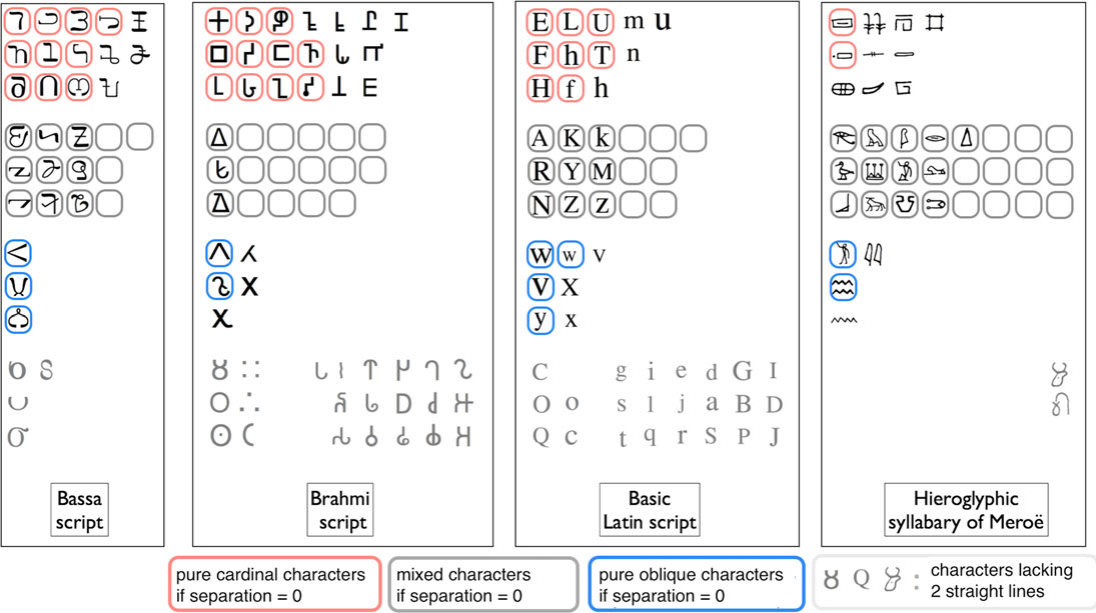
Four scripts illustrating the separation of cardinals and obliques inside letters. Boxed spaces represent the number of pure cardinal, mixed, and pure oblique characters that chance would predict.

For each script, a “separation index” was computed. It measured the extent to which cardinal and oblique lines are mixed between letters or separated. That index is positive for scripts like the Latin script, which have more pure cardinals (E, F, H…) and pure obliques (V, W, X…) than chance would predict (Fig. 1). For each letter in every script, the index takes the chance probability of obtaining a 100% cardinal or 100% oblique letter. It is equal to

$$c^{n}+{\left(1-c\right)}^{n}$$

where *c* is the script's average cardinality, and *n* the number of straight lines in the letter. This index simulates what would happen if one preserved the script's cardinality and the number of straight lines in each letter but allocated a random pick of cardinal and oblique lines to each letter. The average per letter of this index is then subtracted from the actual proportion of pure characters in every script. The result is the separation index. A separation index of 20% means that “pure” letters like E or W are more common than chance would allow by 20 percentage points (e.g., there should be 40% of them, but there are 60%), and “mixed” letters (like K) less so by 20 percentage points. Absolute agreement between raters was acceptable (ICC = 0.760, *n* = 115), like the agreement between Unicode and manuscript sources (ICC = 0.822, *n* = 20). One script, Thaana, had to be excluded because none of the letters in it have more than one straight line.

### 2.3 Vertical symmetry dominance

For each script, two coders counted the number of horizontally or vertically mirror‐symmetrical letters (excluding other types of symmetry). The prevalence of vertical‐symmetrical letters among those mirror‐symmetrical letters was computed for all the scripts that featured more than three usable letters for one coder at least (this cut‐off point was pre‐registered before the measures were taken). The prevalence of vertical symmetry could be computed for 82 scripts from all seven families. Inter‐rater agreement was high (ICC = 0.97, *n* = 82 scripts), as well as agreement between Unicode and manuscript sources (ICC = 0.92, *n* = 18 scripts).

## 3 Results

### 3.1 Cultural selection

The status of each script (live or extinct) was coded using the authoritative literature. This live/extinct variable was then used as predictor in a nested regression analysis. The scripts were grouped into seven families based on the classifications most common in the literature (SM 2.3.), as a way of keeping Galton's problem in check (Mace & Pagel, 1994). These families were used as the grouping variable in linear mixed models with random intercept, using the lme function of the nlme package for R (Pinheiro, Bates, DebRoy, & Sarkar, 2017). A null model was built first, with a random intercept for script family; a second model introduced the script's live or extinct status as a fixed effect. Models were fitted using restricted maximum likelihood, unless indicated otherwise. All reported *p* values are two‐tailed.

### 3.2 Cardinality

**Figure 2:**
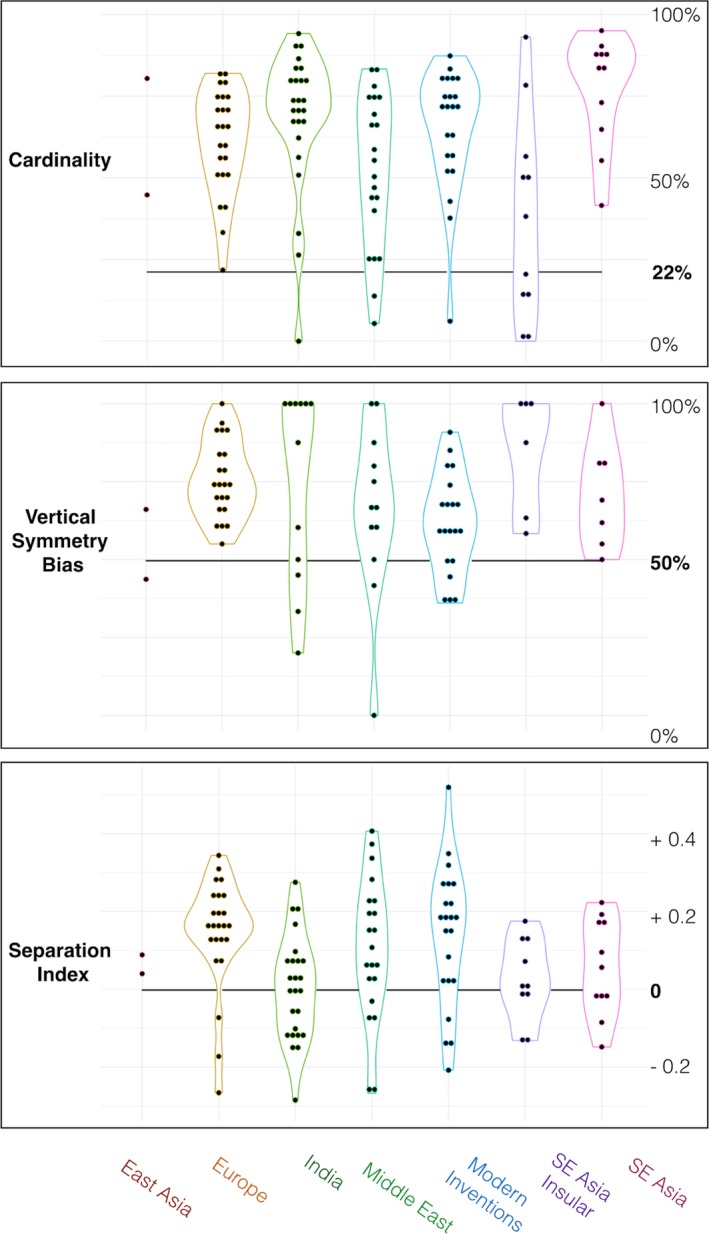
The three legibility features: Cardinal dominance, Vertical symmetry dominance, and Separation, compared to chance levels, in seven families of scripts. Each dot stands for one script. Chance levels (bold face) indicated by full lines.

The null model for cardinality has an intercept value of 61% (95% CI: 51% to 70%; *df* = 109, *t* = 12.6, *p* < .000). Cardinality is above the random threshold (22%) in all seven families of scripts, and in 10 of 14 idiosyncratic scripts (modern inventions that cannot be traced back to one single ancestor) (Fig. 2). Both cardinal orientations, horizontals and verticals, are overrepresented (SM 2.5). Adding the scripts’ status (live or extinct) as predictor resulted in a more informative model (Akaike's information criterion—AIC—of −18.3 vs. −11.3 for the previous model). This new model shows extinct scripts to be less cardinal than live ones (β = −9%, 95% CI: −17% to −0.08%, *df* = 108, *t* = 2.1, *p* = .032). (For this last comparison, the two models were fitted using maximum likelihood.)

### 3.3 Separation of cardinals and obliques

The null model estimates the separation index at +8%, clearly above the chance value of 0 (95% CI: +2% to +14%; *df* = 108, *t* = 3.3, *p* < .005). “Pure” letters like E or W are more prevalent than chance would predict, by 8 percentage points on average. The separation index is positive in all seven families and in 11 of 14 idiosyncratic scripts. It is weak in India and South‐East Asia, presumably because these scripts, which used to be written on flexible leaves, tend to avoid angular shapes for motor reasons (Watts, 1994). One outlier was identified: the Loma script from West Africa, with a separation index of 0.52. Removing it does not change the results shown in this document. The second model that included each script's status (live or extinct) was more informative than the null model (AIC = −99 vs. −97 for the null model), and it included a negative effect for being a live script as opposed to an extinct one (β = −5.9%, 95% CI: +0.1 to +11.7%, *t* = −2.01, *df* = 107, *p* = .046). This tendency ran in the opposite direction as predicted, extinct scripts having a *higher* separation index compared to live ones. (For this comparison, the two models were fitted using maximum likelihood.)

### 3.4 Vertical symmetry dominance

The null model estimates vertical symmetry prevalence at 70.6% (95% CI: 64% to 76.7%, *t* = 22.19, *df* = 74, *p* < .00). Vertical symmetry dominates horizontal symmetry in all seven script families, and in 8 of the 12 idiosyncratic scripts for which it could be measured. One outlier was identified: the Old Persian cuneiform syllabary, which has no vertical‐symmetrical letters at all. Removing it does not change the results of this paper. The second model that included each script's status (live or extinct) was less informative than the null model (AIC = −18 vs. −19 for the null model) and included no significant effect for being a live script as opposed to an extinct one (β: +5% (95% CI: −4.1% to 15%, *t* = 1.12, *df* = 73, *p* = .26). (For this comparison, the two models were fitted using maximum likelihood.)

### 3.5 Cultural transformation

Ninety‐three branching‐out events (a descendant forming from an ancestor) were identified (SM 2.4.). For each of the three features of interest (cardinality, separation index, and vertical symmetry index), a distinct linear mixed model was built to predict the direction of changes in these ancestor‐descendant pairs of scripts. For each pair, the ancestor's value on the measure of interest was subtracted from the descendant's value. These differentials were grouped by ancestry: Scripts sharing the same ancestors were nested together. For each of the three features, the best‐fit linear mixed model showed an intercept value that did not significantly depart from zero, and in two cases, I found some support for the null hypothesis (no directionality in the changes between ancestor and descendant).

### 3.6 Cardinality

**Figure 3:**
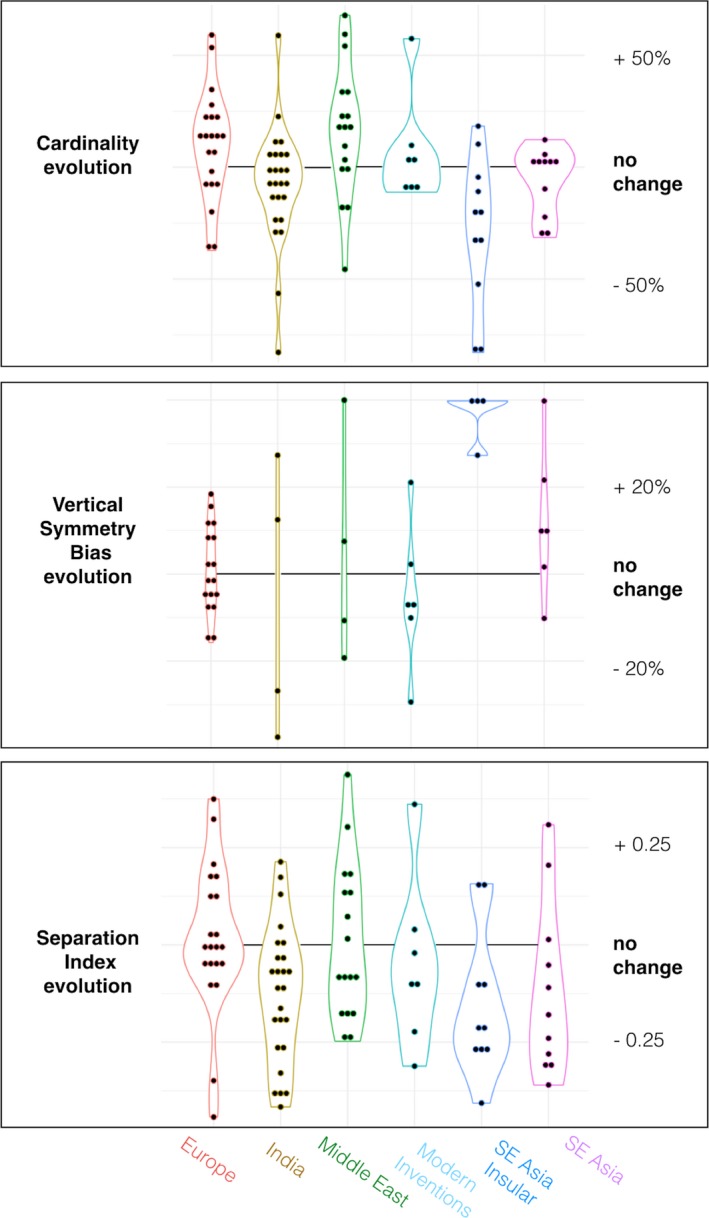
The difference between descendant scripts and ancestor scripts plotted for each documented script in six families (one dot = one script), for three features that affect legibility: Cardinal dominance, Vertical symmetry dominance, and Separation.

The null model indicates a cardinality decrease of less than 1% among descendant scripts compared to their ancestors (95% CI: −9% to +7%; *df* = 65, *t* = −0.17, *p* = .87). Descendants show no tendency to be more cardinal than their ancestors. This lack of a general trend does not make cardinality a highly heritable trait: Descendant cardinality correlates with ancestor cardinality to a weak degree only (Spearman's ρ = 0.30, *p* = .003, *n* = 93 descendant–ancestor pairs). A Bayesian one‐sample *t* test was conducted to see whether the data supported the hypothesis that descendants do not, on average, decrease or increase their cardinality compared to their ancestor. It found moderate support for the null (BF = 3.7) (Fig. 3).

### 3.7 Separation

The null model estimates descendant–ancestor differences in separation index at −0.2% (95% CI: −7% to +6%; *df* = 64, *p* = .95), suggesting no tendency for scripts to expand the separation of oblique and cardinal strokes between letters. Here again, descendant scripts tend not to inherit their ancestor's separation index: the correlation between ancestor and descendant is undetectable (Pearson = 0.03, *p* = .761, *n* = 92 pairs). A Bayesian *t* test similar to the one conducted for cardinality found moderate support for the null hypothesis (BF = 4.7).

### 3.8 Vertical symmetry dominance

The null model estimates descendant–ancestor differentials on this trait at +3.8%, (95% CI: −4% to +12%; *df* = 27, *p* = .37), indicating only a weak increase with time. Vertical symmetry dominance in ancestor scripts was not found to correlate with their descendants’ (Pearson = 0.04, *p* = .78, *n* = 41 pairs). Given the small sample size, the data do not support the null hypothesis (BF = 1.8).

### 3.9 Additional features

As a post hoc analysis (at the editor's invitation), I replicated this result on three additional features of scripts (see SM 2.7). One I considered to be cognitively appealing: the proportion of horizontally or vertically symmetrical letters in the script (on the cognitive benefits of symmetry, see van der Helm, 2015). The other two were neutral features, chosen to prove that my dataset was capable of proving the null hypothesis when it was true. For all three features, the hypothesis of directed transformations was rejected. In each case, the estimated value for the intercept of the linear mixed model includes zero (all *p* > .5), and the Bayes factor indicates moderate support for the null hypothesis (BFs between 3.5 and 4.9).

## 4 Discussion

The results emphasize the cultural impact of flexible, defeasible biases, as opposed to rigid universals (Thompson et al., 2016); at the same time it shows how these biases manifest themselves without relying on the amplificatory powers of cultural transmission. The scripts used in alphabetical or syllabic writing systems are shaped in three ways that make them easier to read. Their cardinality is much higher than chance would predict, they do not let cardinals and obliques mix as much as chance would let them, and vertical symmetry dominates horizontal symmetry among mirror‐symmetrical letters. Cardinal dominance may be (in part) an adaptation to the material bases and processes of writing, like the rectangular page (a possible cause of cardinal dominance in abstract paintings—Miller, 2007), but that is an unlikely explanation for the separation of cardinal and obliques, or the dominance of vertical symmetry. Other studies have found similar effects, where letter shapes tend to reproduce patterns found in the external world (Changizi et al., 2006).

It is worth considering whether the findings presented here can be related to the discriminability of individual letters *within* scripts (Aghababian & Nazir, 2000). Should we expect that the same visual features that make a script visually appealing also make individual letters easier to recognize? Not necessarily. A letter's saliency inside a script depends crucially on non‐visual factors, such as its frequency of use (New & Grainger, 2011) or the reader's expertise with the script (Wiley, Wilson, & Rapp, 2016). Also, appealing visual features may be present in many different letters of the same script (for instance, many Latin letters include a left‐hand‐side vertical bar, which makes them confusable). Such confounds can in theory be controlled away, but this can be difficult with a small dataset (typically, the 26 or 52 letters of the Latin script). My re‐analysis of a classic study in letter perception (Podgorny & Garner, 1979) (see SM 2.10) detected a positive effect of letter cardinality on letter discrimination, in line with what previous studies have shown for the Latin and Arabic scripts (Fiset et al., 2008; Wiley et al., 2016). I also found a tendency for pure letters to be recognized faster than mixed ones (a feature that had not, to my knowledge, been investigated by previous studies). I failed to find a positive effect of vertical symmetry, congruent with previous studies that find no effect of symmetry on letter recognizability (Egeth, Brownell, & Geoffrion, 1976; Wiley et al., 2016). Thus, while the letter recognition data concur with the cultural data as far as cardinality and separation are concerned, vertical symmetry presents an interesting contradiction. Symmetry (vertical or otherwise) has not been proven to make a difference to individual letter recognition in two alphabets, yet the vast majority of scripts include several symmetrical letters, most of them vertically rather than horizontally symmetrical.

The fit documented here between letters and visual constraints is compatible with three scenarios, one in which our three legibility features are a vestige from a pictographic stage in the history of writing, one in which they arose in the course of long‐term cultural evolution, and one where cardinal dominance, separation, and the vertical symmetry bias spontaneously appear in the same short time frame where scripts are invented.

Our three features are unlikely to be a mere legacy of a pictographic origin—vestiges from ancestral iconic scripts. That scenario would fail to explain why these precise visual properties were conserved, even though iconicity itself was abandoned (only one of our 116 scripts—the hieroglyphic alphabet of Meroë—is iconic). It fails to explain why our three features show in most idiosyncratic scripts, many of which have no clear pictographic past. It does not square with the fact that legibility does not decline when new scripts originate from their ancestors. It is also worth noting that none of the three features documented here are heritable to an important degree. This failure of cultural inheritance to preserve basic visual features like cardinality contrasts with high fidelity in other domains. Direction of reading (left to right, right to left, or top to bottom) is a case in point. It is highly preserved, with more than 95% of scripts staying in line with their ancestor (SM 2.6; note that inheritance is maximally conservative for scripts written in the most natural way, from left to right). Thus, something beyond mere inertia seems to be sustaining our three features: They keep being reinvented, with little contribution from cultural inheritance.

An evolutionary scenario is often deemed more likely. In this view, writing systems would have been selected by cultural evolution to fit cognitive constraints. Two indicators of cultural evolution dynamics were examined here: selective extinction, and transformations between ancestor scripts and descendants. Selective extinction shows a small but significant effect for cardinality, not for the other two features. No clear indication was found that scripts branching out from older scripts rate more highly on the aspects of legibility examined here. To be precise, the data provided moderate support for the null hypothesis in three of four relevant cases (cardinality, separation, and symmetry), not for the fourth one (vertical symmetry dominance). The dataset is not lacking in historical depth: It covers at least three millennia—most of the recorded history of writing. How could this apparent weakness of evolutionary trends be explained?

The shape of letters is subject to two competing dynamics of attraction: motor constraints and visual constraints. Writers should try to minimize the effort involved in producing letters, but in so doing they risk a diminution in legibility. Faster hand‐writers produce less legible letters (van Drempt, McCluskey, & Lannin, 2011), as do tired hand‐writers (Parush, Pindak, Hahn‐Markowitz, & Mazor‐Karsenty, 1998). Some aspects of writing appear to have evolved in a manner consistent with motor constraints, but not necessarily with legibility. For instance, rapid handwriting is blamed for the fact that letters tend to lose their distinctive features through cursivization (Parkes, 2008). Such a drift of the Latin script created mirror‐shaped letters (e.g., “p” and “q,” from “P” and “Q”) that our visual system is ill‐equipped to discriminate (Pegado, Nakamura, Cohen, & Dehaene, 2011; Wiebelt, 2004). In that case, a motor constraint distorted the shape of scripts to make them less legible. Another way in which visual constraints may be at odds with motor constraints is script standardization. A handwritten letter is recognizable to the extent that it resembles its prototype: Variability in letter formation is a key characteristic of poor handwriting, and it diminishes legibility (Di Brina, Niels, Overvelde, Levi, & Hulstijn, 2008; Graham, Struck, Santoro, & Berninger, 2006; Longstaff & Heath, 1997). The need to keep one's letters standardized is a visual constraint (it bears on letters’ legibility) and a conservative one: It pushes hand‐writers to keep close to a letter's prototype, as shared by the community. Motor constraints can counteract this pressure, producing variation. As variation is what feeds cultural evolution, we would expect the historical evolution of scripts primarily to reflect motor constraints. This would explain why well‐known evolutionary trends, like cursivization, are detrimental to legibility.

The shape of scripts tends to be determined by a small number of highly prestigious innovators: For more than 20% of our scripts, we know that they had one inventor (a distinct one for each script), and that they changed little since their invention. It has been claimed that the first writing systems were developed by small communities of specialists over short time spans, perhaps no more than one generation (Houston, 2004). This is not a favorable condition for cumulative evolution to take place (Derex, Beugin, Godelle, & Raymond, 2013). Neither common descent nor evolution over the last three millennia seems likely to account, on its own, for cardinal dominance, the separation of cardinal and oblique strokes between letters, and the dominance of vertical symmetry. All three can be observed in independent modern inventions. This compels us to consider another possible source of structure: a general tendency of the human mind to respect sophisticated constraints making for attractive shapes. Cultural adaptation to cognitive preferences may not need long‐term cultural evolution.

## Supporting information


**Online Supplementary Material 1.** The complete dataset on all the “ISO 15924” scripts included in the study is accessible at this address: https://osf.io/8hgr5/.
**Online Supplementary Material 2.** Methodological appendix. https://osf.io/8hgr5/.
