# Supplementary material for: Spontaneous Emergence of Legibility in Writing Systems: The Case of Orientation Anisotropy
Source: Cogn Sci. 2017 Oct 10;42(2):664–77. doi: 10.1111/cogs.12550 (PMC5887916; doi:10.1111/cogs.12550)
Supplement: Supplementary file 1 — Online Supplementary Material 1. The complete dataset on all the “ISO 15924” scripts included in the study is accessible at this address: https://osf.io/8hgr5/. Online Supplementary Material 2. Methodological appendix. https://osf.io/8hgr5/. [file COGS-42-664-s001.docx]

**The spontaneous emergence of legibility in writing systems: cardinal dominance and anisotropic symmetry**

**Online supplementary materials 2: Methodological appendix**

Olivier Morin

**morin@shh.mpg.de**

**Max Planck Institute for the Science of Human History, Jena**

**2.1. Script and letter selection:** Scripts were taken from the list established by the ISO consortium (ISA 15924, consulted in 2013). Logographic or logophonetic systems were excluded, as well as secondary writing systems (like the phonetic alphabet or the Yi script, that do not stand on their own but accompany another system), non-visual writing systems (like Braille), writing systems that do not directly encode a spoken language (like stenographies), and systems that were invented as part of a work of fiction. The full list of exclusions can be found in section 2.12. Inside scripts, only letters that could stand on their own to indicate a sound were included: punctuation marks, ligatures, diacritic marks (including those used to indicate vowels in abjads), number symbols, honorific marks, currency marks and other ideographic symbols, were not included. In writing systems with a plurality of cases (e.g. majuscules and minuscules), all cases were measured. For 6 syllabaries, the inventory of characters was so large that only a portion (never less than a third of the characters, randomly selected using the ending of their Unicode serial number) was coded (they are signaled in the SM 1).

Unicode provides standardized versions of my 116 scripts. They are meant to enable the digital treatment of contemporary or ancient writings: the characters are modeled on handwritten or printed material, but they are meant to be typed, not handwritten. They are ideal material to investigate the impact of visual (as opposed to motor) constraints on letter shape. The study considered only characters from alphabetic, syllabic and alphasyllabic systems, as characters in logographic and logo-syllabic systems (a clear minority of scripts) were too numerous and, above all, too complex to be analyzed.

**2.2. Coding and data collection**: The coding was done by the first author and, independently, by a coder unaware of the aims of the study. As a result of Unicode standardization, all letters were presented in boxes of identical sizes.

For the purpose of measuring cardinality and separation, arbitrary criteria were set to define what qualifies as a straight line. Reported lines needed either to be of a given length L (at least) and cut off from the rest of the letter by a sharp turn or a void, and straight, or to be of length 2xL (at least), and straight. Unicode standardized letters were always measured on a MacBook pro screen with a resolution of 1280x800 pixels, and the document was enlarged with a 1600% zoom. Length L was fixated at 80 pixels. Length and orientation were measured using PixelStick software, an electronic ruler and compass. Curved shapes (any shape that did not stay along the edge of the ruler was deemed to be curved) were ignored if they did not contain at least one straight line (as defined above). The independent coder had been previously trained to use the tool and the coding system on J.R.R. Tolkien's fictional scripts. Fig. 1 provides an illustrative example of the coding process.


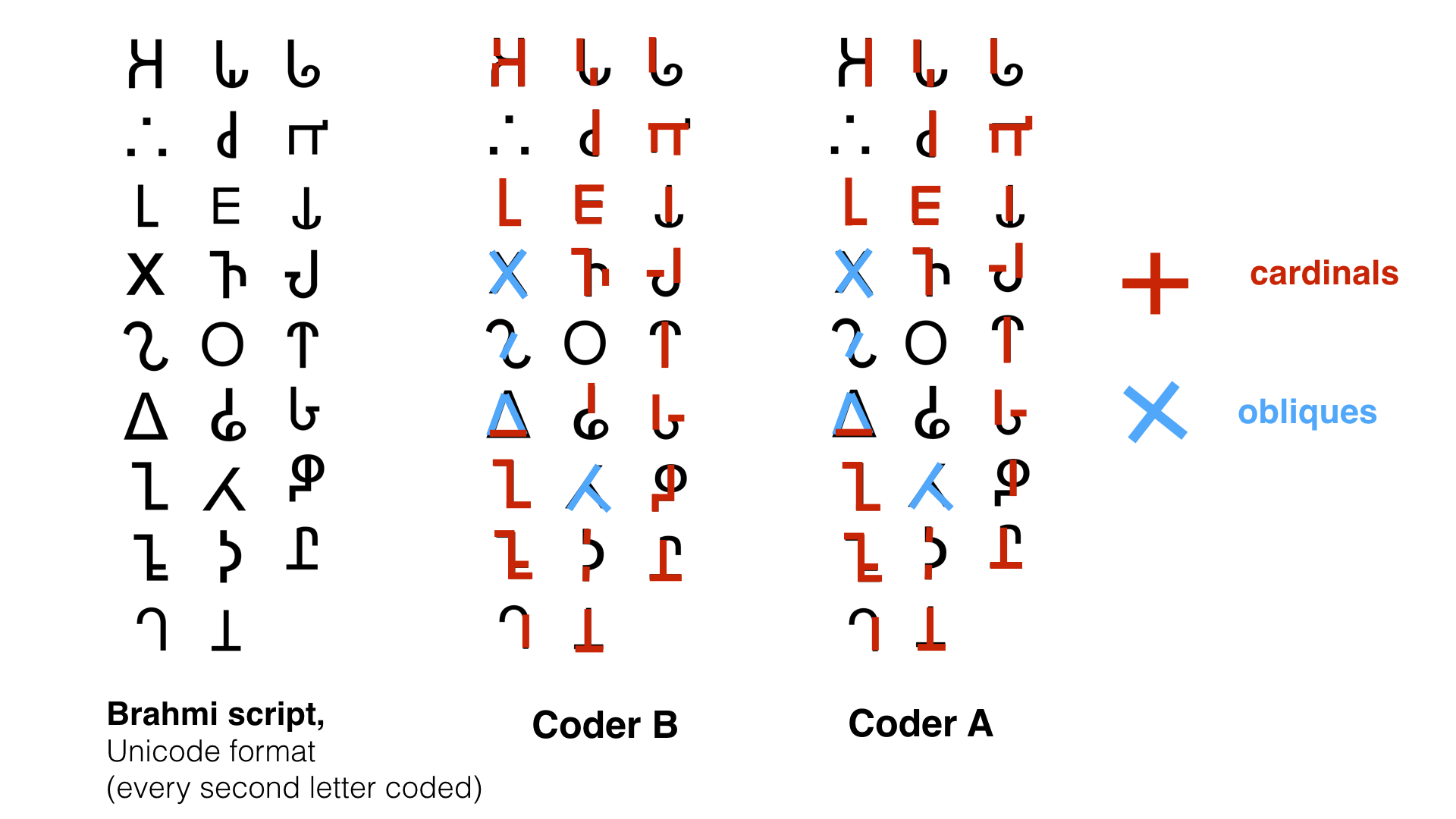


**Fig.1:** An example of the coding process for cardinality: The Brahmi script (Unicode format). To save space, every second letter of the script is shown (in Unicode order). Inter-rater agreement was higher than average on this script.

The coding of vertical and horizontal mirror-symmetry was not formalized in any special way, except for one thing: Because mirror-symmetry is easier to detect when it is vertical, all the letters were rotated 90° for one coder. The decision to consider only scripts with 3 mirror-symmetrical letters or more was taken blindly and before any relevant data were collected, as attested by a preregistration on the open science framework (Morin & Miton 2014; https://osf.io/ydkmw/). The data were collected in two distinct phases, as the paper underwent revisions. The data on cardinality and oblique/cardinal lines separation were collected in 2014 (before the authors took the habit of keeping an online preregistered record of data collection) while the data on symmetry were collected in 2016 (which is why only a part of the study is preregistered).

**2.3. Scripts classification:** The scripts were classified into seven families, on geographic and phylogenetic grounds. This classification corresponds in many ways to that proposed by standard references (Daniels & Bright, 1996) (see the list of sources in section 2.13 of this document).

*- Middle Eastern family:* direct descendants of the scripts of the Middle East: Egyptian, Cuneiform, South Arabic, and Aramaic.

*- Phoenician family:* all the direct and indirect descendants of the Phoenician alphabet, including Greek and its descendants.

*- Indian Brahmic family:* all the descendants of the Brahmic script in Modern India, Pakistan, Sri Lanka, Mongolia and Tibet

*- Mainland South-East Asian Brahmic family:* all the direct and indirect descendants of the Brahmic script outside in mainland South-East Asia.

*- Insular South-East Asian Brahmic family:* all the direct and indirect descendants of the Brahmic script outside in Indonesia and the Philippines.

*- Recent inventions family:* all the scripts created after 1800 have been gathered into one family, following widespread usage (Daniels & Bright, 1996), since they share many properties. Few of them are evolutionary derivations of a single script: most are idiosyncratic inventions or fusions of several scripts. All but one (G.B. Shaw's Shavian) were invented in a colonial or post-colonial context.

*- East Asian family:* Korean Hangul and Japanese Kanas.

**2.4. Phylogenetic analysis and interpretation of phylogenetic trends:** Each script's last consensual common ancestor was determined by pooling together various sources (see SM 1 for the complete list of sources). When different sources gave contradictory information, a majority rule was applied. When sources were consistent with one another but differed in their specificity, the most specific source (citing the ancestor that was closest in time to its descendant) was chosen. For instance, if one source cited the Brahmic script as the ultimate ancestor of the Gurmukhi script, and another cited the (Brahmi-derived) Sarada script, the latter source was chosen.

**2.5. Horizontals and verticals:** To make sure that cardinal dominance was not purely a "verticality" or "horizontality bias", the average proportion of vertical and horizontal line per letter is reported for each script (absolute agreement between coders: ICC = 0.965 for horizontality, 0.979 for verticals). Since lines were counted as horizontal or vertical if they lied within a 10% distance of the 90° and 180° axis, the chance prevalence for each of the cardinal directions is 11%. Average horizontality for my scripts is 23.7% (SD 16.2 %), average verticality is 37.7% (SD 22 %). All 7 families are above the chance threshold for horizontality (M = 24 %, chance threshold: 11 %) and verticality (M = 38 %, chance threshold: 11 %). The 14 idiosyncratic scripts, likewise, are above the thresholds for horizontality (M = 20 %) and verticality (M = 43 %). The strong tendency for vertical lines to outnumber horizontals is consistent with the view that horizontal lines are more difficult to produce, from a motor point of view, than horizontals (Meulenbroek & Thomassen, 1991).

**2.6. Directions of reading.** The usual direction of reading and writing for each of the scripts in the sample was determined from the literature without any double coding, since my sources overwhelmingly agree on this matter. One script (Old Church Slavonic) does not have one clear direction (and is often written in boustrophedon style), and it wasn’t considered. Overall, a Left-Right direction of writing and reading was clearly dominant, as could be expected from the fact that most people are right-handed. 90 scripts are LR (left to right), 23 are RL (right to left), while 2 are usually read from top to bottom (TB). All of the 14 “idiosyncratic” scripts are LR. Direction of writing is conservative: descendant scripts tend not to switch directions—this happens only in 9 cases, for 91 ancestor-descendant pairs. Even descendants of RL scripts will usually be RL as well, in spite of the low baseline probability of opting for an RL script (see Table 1). Interestingly, direction is much more faithfully inherited when the ancestor is LR. There are 3 switches for 64 scripts with an LR ancestor, vs. 6 switches for 27 scripts with an RL ancestor. This difference is statistically significant (chi-square: 6.55, df = 1, p = 0.01, 2-tailed). Note that in the case of the Mende script, which is an RL script in spite of having an LR ancestor, the R-L direction of writing most probably comes from Arabic, which was the second most important influence on the script after the Vai syllabary.

| Ancestor’s direction | LR scripts | RL scripts | TB scripts |
| --- | --- | --- | --- |
| LR | 61 | 2 | 1 |
| RL | 5 | 21 | 1 |
| No ancestor in the dataset  (idiosyncratic scripts +  ancestors not studied in this dataset) | 24 | 0 | 0 |
| Total | 90 | 23 | 2 |

Table 1. Habitual direction of reading and writing in 115 scripts.

**2.7. Additional features: Size, Straightness, and Symmetry**

I observed the transformations between ancestors and descendants for 6 features in all. In addition to my three main features of interest (cardinality, vertical symmetry dominance, and separation), the editor recommended I add other features, including “neutral” features for which no evolutionary trend was predicted or likely. All three were added in a late revision of the paper, after the main analysis had been conducted.

The features chosen were (i) Symmetry—the proportion of mirror-symmetrical letters in a script; (ii) Straightness—the average number of straight strokes per letter, and (iii) Size—the number of letters in a script. While Symmetry is cognitively appealing, I considered Script size and Straightness to be neutral features, i.e. not particularly appealing from a functional or cognitive point of view. The features are further described below.

**Symmetry**. Two coders counted the proportion of mirror-symmetrical letters (i.e., horizontally or vertically symmetrical letters) in each script (ICC = 0.94). 90% of scripts contain at least one mirror-symmetrical letter.

**Straightness**. The average number of straight strokes per letter could be straightforwardly taken from my previous analyses. 10 scripts were chosen at random to compute the inter-rater reliability between coder A and coder B. The ICC was reasonably high: 0.93 (95% CI: 0.66 to 0.98), so that it was decided to use only coder A's data for the resulting analysis.

**Size**. A script's size was defined as the number of characters typically used together by the community that uses the script (“script size”). For some scripts, the inventory of characters involved characters that were not used simultaneously — for instance when a script is shared between two languages, one linguistic community uses characters unknown to the other one. For such scripts, only the size of the most standard version (as provided by Daniels & Bright 1996) was considered (for instance, the Unified Canadian Aboriginal Script was considered to be 105 characters large, the size of the standard Cree syllabary) The two East Asian scripts were not included in these analyses, since they are typically used in combination with Chinese characters, meaning that the number of characters in those two scripts will underestimate the number of letters that each character can be confused with. This exclusion did not change the pattern of significant results reported here and in the main text.

For each trait, I considered the descendant-ancestor differential, obtained by subtracting the ancestor’s value from the descendant’s value on the trait of interest. This trait was modeled using a linear mixed model, fitted by Restricted Maximum Likelihood, and using two grouping variables: (i) ancestor and (ii) script family. Table 2 shows the β value for each trait. The values for the first 5 traits are presented in decimal format (i.e., -0.007 for the cardinality differential means that descendants are less cardinal than ancestors by 0.7 percentage points). In all six cases the confidence interval includes the value zero (Figure 2).

| **Trait** | **Descendant-ancestor difference (null model’s β for intercept)** | **95 % confidence interval** | **t-value** | **Degrees of freedom** | **p-value (2-tailed)** | **N**  **(scripts /ancestors)** |
| --- | --- | --- | --- | --- | --- | --- |
| **Cardinality** | **-0.007** | **-0.087 to +0,073** | **-0.169** | **65** | **.87** | **92 / 27** |
| **Separation** | **-0.002** | **-0,068 to +0,064** | **-0.065** | **64** | **.95** | **92 / 27** |
| **Vertical Symmetry dominance** | **0.038** | **-0,044 to +0,12** | **0.91** | **27** | **.37** | **41 / 14** |
| **Symmetry** | **- 0.021** | **-0,091 to +0,049** | **-0.612** | **65** | **.54** | **92 / 27** |
| **Straight strokes per letter** | **0.033** | **-0,223 to**  **+ 0.289** | **0.259** | **65** | **.79** | **92 / 27** |
| **Script size** | **- 1.75** | **-8.93 to +5.43** | **-0.487** | **65** | **.63** | **92 / 27** |

**Table 2.** The difference between ancestors and descendants for the six traits of interest, as assessed by six linear mixed models.


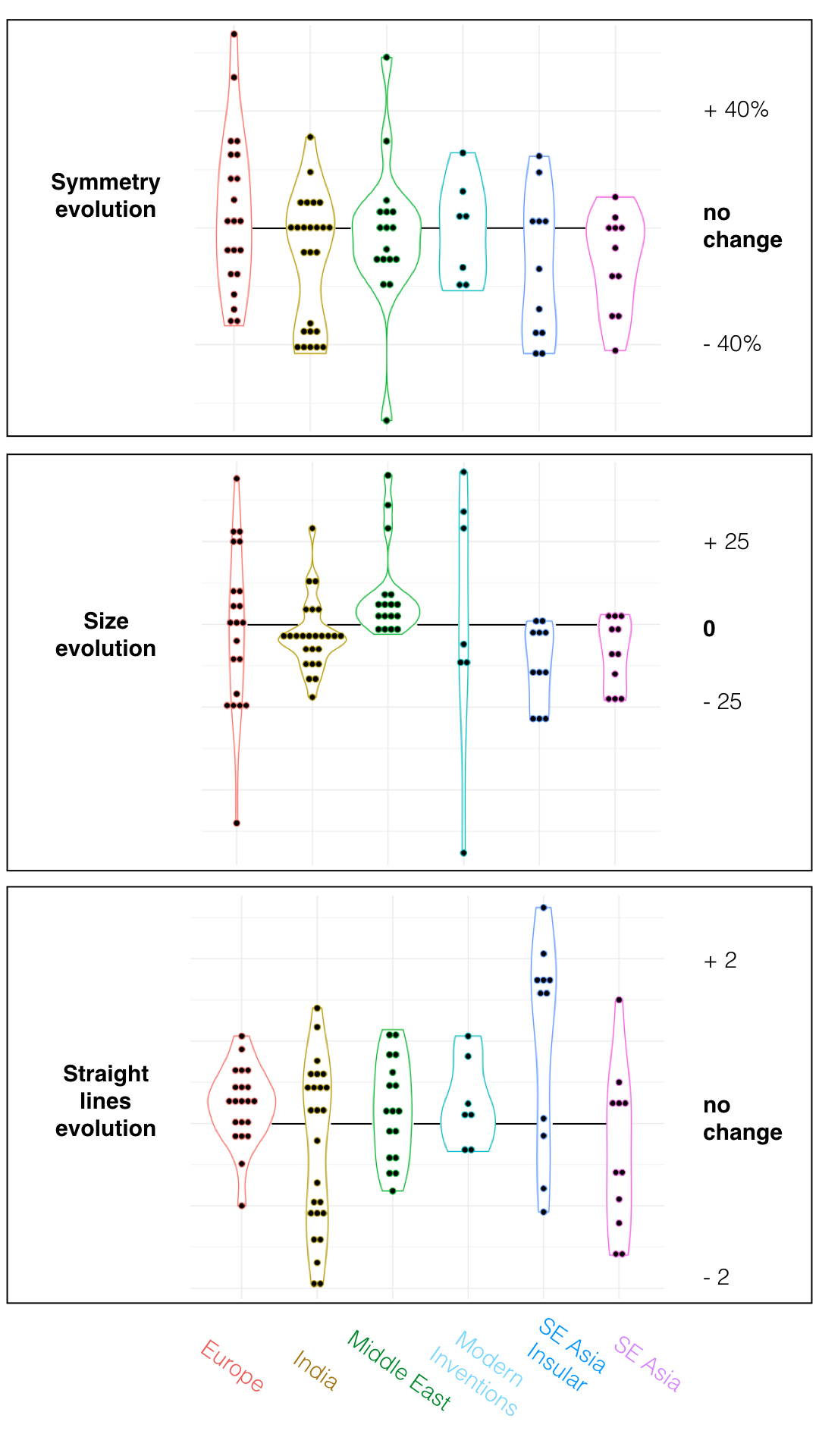


**Figure 2**. The difference between descendant scripts and ancestor scripts plotted for each documented script in six families (one dot = one script), for three features of interest: The proportion of horizontally or vertically symmetrical letters, the script’s size, and the average number of straight lines per letter.

I also sought to replicate my observation that cultural selection does not have an important influence on the morphology of scripts (a script’s being live rather than extinct increases its cardinality somewhat, but does not help with separation or vertical Symmetry dominance). The analysis replicated the one in the main paper. I modeled the three traits­—size, symmetry, straightness,—first by means of a null model (using script family as grouping variable), then with a second model adding a fixed effect for the script’s live/extinct status. A script’s being extinct correlated with slightly *higher* symmetry (β: - 6%, 95% CI: -0,8% to 12,8%) but the effect was only tendentially significant (p = .08) although the second model was more informative than the first (AIC of -62 vs. -60). Regarding size and straightness, the Live/Extinct variable made no significant difference (AIC higher for the second model, both p-values for fixed effect > .5).

**2.8. Bayesian analysis: Support for the null hypothesis**

Our results suggest that the null hypothesis may be true (no tendency for descendants to diverge from ancestors in a particular direction); but the linear mixed effects model analyses so far presented do not test it directly. I computed the Bayes Factor for the null, using a Bayesian t-test (ttestBF function in the BayesFactor package for R — Morey, 2015). The t-test was a one-sample t-test, which took, as data units, each of the ancestor scripts. The value of each ancestor was the average descendant-ancestor differential for that ancestor and for the trait of interest. I took the reciprocal of the Bayes Factor for the t test (1/BF) as a measure of support for the null hypothesis (Rouder, Speckman, Sun, Morey, & Iverson, 2009).

Results indicated moderate support for the null hypothesis for every trait except vertical symmetry dominance (BF = 1.8), which showed a (non-significant) tendency to increase in the previous analysis. The Bayes Factor indicated moderate support for the null, concerning Cardinality (BF = 3.7), Separation (BF = 4.7), Symmetry (BF = 3.5), Script size (BF = 4.2), and the number of straight strokes per letter (BF = 4.9).

**2.9. Are changes in size tied to changes in legibility?**

Since cardinal dominance, separation of obliques from cardinals, and the dominance of vertical symmetry are all likely to enhance the visual discriminability of letters, I predicted that they should increase with the number of characters in a script. Later, I tested this prediction for symmetry as well. A large script is more likely to contain confusable letters; additionally, characters will be less frequent and therefore less recognizable in a large script (Balota, Yap, & Cortese, 2006). Thus, the pressure for legibility should increase as scripts grow.

For each of the traits of interest already considered (script size and straightness excepted), I considered whether my model of descendant-ancestor differentials could be improved by adding the size differential between descendant and ancestor as a predictor. I hypothesized that letters in larger scripts need to be more legible than letters in smaller scripts. Therefore, the four features (cardinal dominance, separation between cardinals and obliques, vertical symmetry dominance, and the prevalence of mirror-symmetrical letters) should become more pronounced in scripts as scripts grow. This hypothesis was only verified for one trait: Cardinality. Scripts were more likely to grow in cardinality, compared to their ancestor, when they also grew in size. The estimate for the effect of descendant-ancestor size differential was moderate but significant (β = + 3,3%, 95% CI: +0,5 to +6,1%, t = 2.3, p = .024). A comparison of this model against the null showed it to be more informative (AIC = 15 vs. 19 for null model). The same comparison applied to the other four traits of interest did not show any substantial contribution of the descendant-ancestor length differential in determining the trait’s evolution (table 4).

| Descendant-ancestor differential for… | AIC for null model | AIC for model with size differential | Model comparison (ANOVA): p-value |
| --- | --- | --- | --- |
| **Cardinality** | 19.9 | 17.3 | .031 |
| **Separation** | -70.0 | -68.9 | .337 |
| **Vertical Symmetry dominance** | -15.2 | -13.8 | .451 |
| **Symmetry** | -28.6 | -26.7 | .77 |

**Table 4**. The effect of including descendant-ancestor size differentials in models of the evolution of traits affecting legibility. (For the purpose of this comparison, the models were fitted using maximum likelihood.)

**2.10. Re-analysis of Podgorny & Garner (1979)**

As a post-hoc analysis (following the editor's recommendation), I reanalyzed the data from an authoritative experiment on letter discriminability (Podgorny & Garner, 1979—experiment 1), to test whether the three features of interest for this study—cardinality, separation, and vertical symmetry—made a difference to the discriminability of Latin letters. Podgorny and Garner asked their subjects to make a same-different judgment between pairs of letters, and reported reaction times for both types of stimuli (“same” pairs and “different” pairs). For any given letter, averaging the reaction times for every pair in which it figures provides a proxy for the letter’s recognizability.

Each of the three visual features of interest were estimated by me based on the paper's Figure 1 (Podgorny & Garner's experiment used, in 1979, a digital display that was quite different from today’s displays). As the authors did, I averaged the subjects' reaction times, for each letter, across all the contexts where that letter was presented (reaction times were measured in milliseconds). I used a linear model, fitted using restricted maximum likelihood, to predict this averaged reaction time for each letter. As a control, I used Podgorny & Garner’s own data regarding the perceived similarities between letters, a proxy for each letter’s confusability (Table 2). For each letter, I used the average similarity rating given for the letter when compared to each of the other letters. A higher rating indicated *greater* similarity. A first model predicting reaction times as a function of similarity showed a positive effect of similarity on reaction times (β = -2.26, SE = 0.84, t = -2.699, p = .0125). In other words, it took longer for subjects to recognize the letters they rated as more similar to other letters. Since this confusability variable had substantial predictive power (model’s R-squared = 20%), I kept it for subsequent analyses.

The three features of interest were then tested by considering, for each feature, one model with that variable and frequency as a predictor. The three features were not used together inside one model because multicollinearity was a concern, and because the low n (26 letters) did not allow me to test for many variables.

Cardinality had a negative effect on reaction times. The model that included this variable had a lower AIC than my first model (169 vs. 172) and it included a negative effect of Cardinality (β: -10.2, SE = 4.8, t = 2.10, p = .046, n = 26). In other words, subjects were faster when identifying cardinal letters (controlling for confusability).

Vertically symmetrical letters did not take less time to detect than others, on the contrary. The model that included this variable had a lower AIC than my first model (168 vs. 172) and it included a negative effect of Vertical Symmetry—longer reaction times (β: + 5.9474, SE = 2.4048, t = 2.473, p = .02121, n = 26).

Subjects were slightly faster at recognizing “pure” letters as compared to “mixed” letters. The model that included this variable had a lower AIC than my first model (168 vs. 172) and it included a tendentially negative effect of separation (β: - 4.4448, SE = 2.3188, t = -1.917, p = 0.067, n = 26).

This analysis should be taken with a grain of salt, given that important confounds (chiefly the relative frequency of letters) cannot controlled for, due to the small sample size. It suggests that cardinality makes individual Latin letters more legible, confirming Podgorny & Garner's observation that "the visual property of diagonality (...) appears to be associated with a slow "same" response." (1979: 46). The observation that cardinal lines tend to make a greater contribution to letter recognition than oblique ones has since been replicated for the Latin script (Fiset et al., 2008) and for the Arabic script (Wiley, Wilson, & Rapp, 2016), although in both cases it appears to be driven by horizontal rather than vertical strokes, possibly because vertical strokes are too common to make a difference between letters. The failure to find a positive effect on reaction times for vertical (as opposed to horizontal) symmetry is also consistent with Egeth et al.'s result (Egeth, Brownell, & Geoffrion, 1976), in a paper that considered reaction times in judgments of similarity for vertically symmetrical as opposed to non-symmetrical or horizontally symmetrical letters (see also Wiley et al. 2016 who do not find that mirror symmetry makes individual Arabic letters more discriminable).

**2.11. Credits for the fonts used in figure 2:** Meroitic font courtesy of George Douros; Brahmi font courtesy of Lennart Lopin; Bassa font: Freelang Font Center.

**2.12. Numbered references for S.M.1.** These references, which provided some of the data for the study, are listed in the datasheet of S.M.1. wherever they have been used, and designated by numbers:

**0** ISO 15924 list: <http://www.unicode.org/iso15924/iso15924-codes.html> (last consulted 09/2016)

**1** Rogers, H. (2005), *Writing Systems: a linguistic approach.* Oxford: Blackwell.

**2** Daniels, PT & Bright, W (1996) *The World’s Writing Systems.* NY: Oxford UP.

**3** AncientScripts, consulted 08/2013

http://www.ancientscripts.com/ws_regions.html

**4** Documents from the Unicode corporation: proposals to encode scripts into the Unicode format.

**5** Scriptsource

**6** Wikipedia online encyclopedia

**7** Omniglot, consulted 08/2013

**8** The Ethnologue database, consulted 08/2013

**2.13. List of rejected ISO scripts with motivation for rejection**

This section lists all the scripts (referred to by ISO code) that were not included in the study, giving the reason in each case.

**Bliss**. Ideographic.

**Bopo**. Phonetic alphabet not used independently from other scripts.

**Brai**. Not a static and visual writing system.

**Cham**. Not a single straight line.

**Cirt**. Tolkien-invented script. Used to train coders.

**Dupl**. Ideographic.

**Egyh**. Egyptian Hieroglyphs: consonant-based logography.

**Egyp**. Egyptian Hieroglyphs: consonant-based logography.

**Hani**. Sinograms: logography.

**Hans**. Sinograms: logography.

**Hant**. Sinograms: logography

**Hira**. Already included in the Hrkt category

**Hluw**. Anatolian Hieroglyphs: logography

**Inds**. Undeciphered pictography/logography

**Jpan**. Super-category including Kana and Kanji

**Jurc**. Logographic.

**Kana**. Already included in the Hrkt category

**Kore**. Super-category including Hangul and chinese characters.

**Latf**. Fraktur. Not present in Unicode nor considered for inclusion. Ruled out as a simple typeface of Latin script. Fused with Latn.

**Latg**. Gaelic shorthand. Not present in Unicode nor considered for inclusion. Ruled out as a simple typeface of Latin script. Fused with Latn.

**LinA**. Undeciphered logo-syllabary. No consensus on what counts as the syllabary. Unicode list is very long.

**Maya**. Pictographic glyphs.

**Moon**. Not a static visual writing system.

**Nkgb**. Phonetic alphabet not used independently from other scripts.

**Nshu**. Syllo-logographic. 300+ glyphs.

**Phlv**. Rejected — UNICODE recognizes only inscriptional Pahlavi (Phli).

**Phlp**. Rejected — UNICODE recognizes only inscriptional Pahlavi (Phli).

**Roro**. Rapa Nui undeciphered pictography/logography.

**Sara**. Tolkien-invented script

**Sgnw**. Not a static visual writing system

**Syre**. Estrangelo Syriac Unicode admits only one category for Syriac scripts: fused with Syrc.

**Syrj**. Western Syriac. Unicode admits only one category for Syriac scripts: fused with Syrc.

**Syrn**. Eastern/Nestorian Syriac Unicode admits only one category for Syriac scripts: fused with Syrc.

**Tang**. Logo-syllabary.

**Teng**. Tolkien-invented script

**Visp**. Not a static visual writing system.

**Xsux**. Cuneiform: logography.

**Yiii**. Still not a pure syllabary in spite of reforms (according to Daniels & Bright 1996).

**2.14. References for this document**

Balota, D. A., Yap, M. J., & Cortese, M. J. (2006). Visual word recognition: the journey from features to meaning (A travel update). In *Handbook of Psycholinguistics* (Academic Press, pp. 285–375). New York: Matthew Traxler & Morton Gernsbacher.

Balota, D. A., Yap, M. J., & Cortese, M. J. (2006). Visual word recognition: the journey from features to meaning (A travel update). In *Handbook of Psycholinguistics* (Academic Press, pp. 285–375). New York: Matthew Traxler & Morton Gernsbacher.

Daniels, P. T., & Bright, W. (1996). *The world’s writing systems*. New York: Oxford University Press.

Egeth, H. E., Brownell, H. H., & Geoffrion, L. D. (1976). Testing the role of vertical symmetry in letter matching. *Journal of Experimental Psychology. Human Perception and Performance*, *2*(3), 429–434.

Fiset, D., Blais, C., Ethier-Majcher, C., Arguin, M., Bub, D., & Gosselin, F. (2008). Features for identification of uppercase and lowercase letters. *Psychological Science*, *19*(11), 1161–1168. https://doi.org/10.1111/j.1467-9280.2008.02218.x

Meulenbroek, R. G. J., & Thomassen, A. J. W. M. (1991). Stroke-direction preferences in drawing and handwriting. *Human Movement Science*, *10*(2–3), 247–270. https://doi.org/10.1016/0167-9457(91)90006-J

Morey, R. (2015). *BayesFactor package for R* [R]. Retrieved from http://bayesfactorpcl.r-forge.r-project.org/

Podgorny, P., & Garner, W. R. (1979). Reaction time as a measure of inter- and intraobject visual similarity: Letters of the alphabet. *Perception & Psychophysics*, *26*(1), 37–52. https://doi.org/10.3758/BF03199860

Rouder, J., Speckman, P., Sun, D., Morey, R., & Iverson, G. (2009). Bayesian t-Tests for Accepting and Rejecting the Null Hypothesis. *Psychonomic Bulletin & Review*, *16*, 225–237.

Wiley, R. W., Wilson, C., & Rapp, B. (2016). The effects of alphabet and expertise on letter perception. *Journal of Experimental Psychology: Human Perception and Performance*, *42*(8), 1186–1203. https://doi.org/10.1037/xhp0000213
